# Median Nerve Palsy following Elastic Stable Intramedullary Nailing of a Monteggia Fracture: An Unusual Case and Review of the Literature

**DOI:** 10.1155/2011/682454

**Published:** 2011-04-20

**Authors:** Surjit Lidder, Nima Heidari, Florian Amerstorfer, Stephan Grechenig, Annelie M. Weinberg

**Affiliations:** ^1^Department of Trauma and Orthopaedics, Royal London Hospital, London E1 1BB, UK; ^2^Department of Paediatric and Adolescent Surgery, Medical University of Graz, Auenbruggerplatz 34, 8036 Graz, Austria; ^3^Department of Traumatology, Medical University of Graz, Auenbruggerplatz 7a, 8036 Graz, Austria

## Abstract

Monteggia fractures are rare in children, and subtle radial head dislocations, with minor plastic deformation of the ulna, may be missed in up to a third of cases. Complications of Monteggia fractures-dislocations include persistent radial head dislocation, forearm deformity, elbow stiffness, and nerve palsies at the time of presentation. An unusual case of median nerve palsy following elastic stable intramedullary nailing of a type I Monteggia lesion in a 6-year-old girl is presented, and we highlight that, although most nerve palsies associated with a Monteggia fracture-dislocations are treated expectantly in children, early intervention here probably provided the best outcome.

## 1. Introduction

Monteggia fractures are rare and account for 0.4% of all forearm fractures in children [1]. Bado type I Monteggia lesions, with the radial head dislocated anteriorly, are the commonest and account for up to seventy percent of these injuries in children. However subtle radial head dislocations, with minor plastic deformation of the ulna, may be missed in up to a third of cases [2, 3]. Complications of Monteggia fractures-dislocations include persistent radial head dislocation, forearm deformity, elbow stiffness, and nerve palsies at the time of presentation [2, 4–7]. 

We present a case of median nerve palsy following elastic stable intramedullary nailing (ESIN) of a type I Monteggia lesion in a 6-year-old girl. To our knowledge, there have been no previous reports of such an injury in the orthopaedic literature. Intraoperative iatrogenic nerve injuries as a result of ESIN are extremely rare. We highlight that although most nerve palsies associated with Monteggia fracture-dislocations are treated expectantly in children, early intervention here probably provided the best outcome.

## 2. Case Report

**Figure 1:**
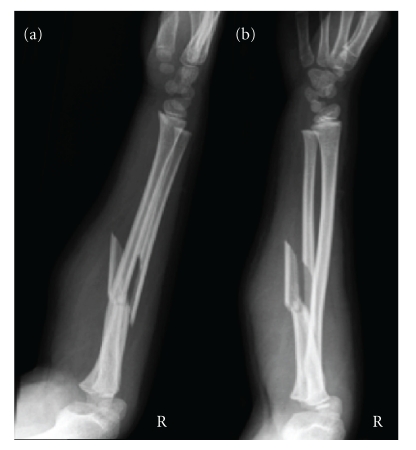
Lateral (a) and oblique (b) radiographs of a right type I Monteggia fracture-dislocation.

A six-year-old right hand dominant girl was referred to our institution after jumping off a swing and falling on to her outstretched right arm. Initial examination revealed a swollen and deformed forearm, ecchymosis over the elbow and midforearm, and a limited range of elbow movement. There was no distal neurovascular deficit. Radiographs showed a Bado type I Monteggia fracture-dislocation (Figure 1).

**Figure 2:**
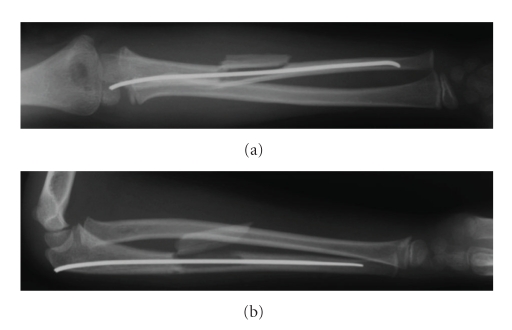
Postoperative anteroposterior (a) and lateral (b) radiographs of the right forearm with a 2 mm titanium elastic nail in the ulna. Diaphyseal ulna fragment not reduced.

Manipulation under general anaesthesia and percutaneous fixation of the ulna with a 2 mm titanium elastic nail was performed due to the unstable ulna diaphyseal fracture. An intraoperative decision by the senior author was made not to manipulate and reduce the displaced ulna fragment as it did not interfere with forearm rotation and the potential for remodelling in this six-year-old child (Figure 2). A lightweight splint was applied with the elbow flexed at ninety degrees and the forearm in full supination. The patient was discharged home the following day, comfortable and with no neurovascular deficit. 

At review on day ten, the patient complained of new pain over the volar aspect of the right forearm and paraesthesia in the right thumb and index finger. Clinical examination revealed resolving ecchymosis, sensory deficit in the median nerve distribution, and painful flexion of the wrist. Electromyography confirmed median nerve neuropathy, and magnetic resonance imaging of the right forearm showed contusion of the median nerve with the distal spike of the ulna fragment abutting the nerve.

**Figure 3:**
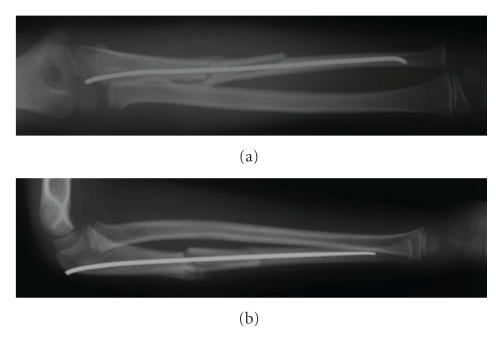
Anteroposterior (a) and lateral (b) radiographs of right forearm two weeks after exploration.

**Figure 4:**
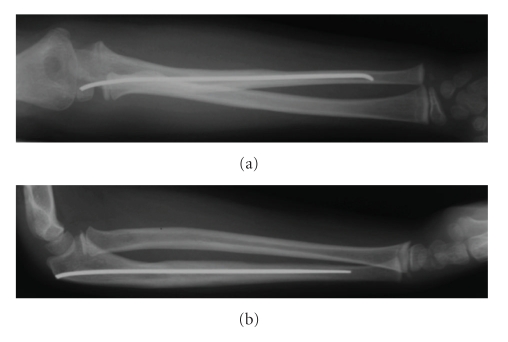
Anteroposterior (a) and lateral (b) radiographs of right forearm at six months showing fracture union and remodelling.

It was decided to surgically explore the median nerve as the delayed development of pain and median nerve symptoms indicated progressing nerve compression. The median nerve was identified as it passed deep to the fibrous arch of the flexor digitorum superficialis muscle. It is accompanied by the ulna artery here. The median nerve was abutting against and stretching over the large butterfly fragment from the ulna. The median nerve was freed, and soft tissue interposition between the ulna butterfly fragment and the shaft was cleared. The butterfly fragment was then reduced and secured using vicryl sutures through the periosteal sleeve to the shaft. Immediately postoperatively the patient no longer suffered from the neuropathic forearm pain. At two-week follow-up, the patient remained pain-free and no longer exhibited a neurological deficit. Check forearm and elbow radiographs demonstrated maintained reduction of the radial head, good position of the ulna diaphyseal fragment (Figure 3), and at six months the fracture had united and remodelled (Figure 4). 

**Figure 5:**
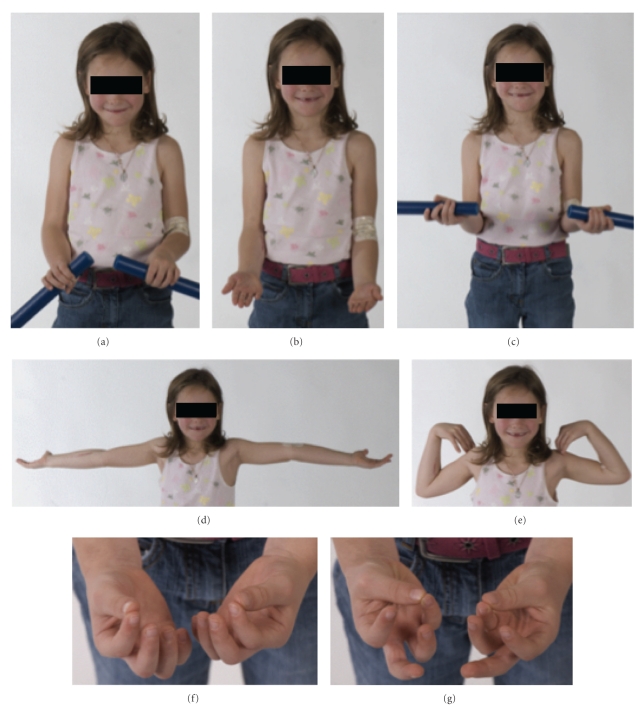
Clinical photographs demonstrating range of movement at nine months of the forearms in pronation (a), supination with flexion of 45 degrees (b) and 90 degrees (c), elbow extension (d), elbow flexion (e), and full median nerve function (f, g).

The elastic stable intramedullary nail was removed at nine months from the index injury. The patient continued to be asymptomatic with no motor or sensory deficit of the median nerve and demonstrated excellent clinical function with full flexion and extension at the wrist, active forearm rotation consisting of full supination, 50° of pronation and full flexion, and extension at the ulnohumeral joint (Figure 5).

## 3. Discussion

Monteggia fractures in children are rare and occur between the ages of 4 to 10 [1]. Bado originally classified Monteggia fracture-dislocations into true Monteggia lesions (type I–IV) (Table  1) and equivalent lesions [8]. In type I, the radial head is dislocated anteriorly with a concomitant ulna diaphyseal fracture. Often subtle radial head dislocations are missed. On a true lateral radiograph of the elbow a line drawn through the radial neck and head passes through the centre of the capitellum with alignment maintained throughout the full range of elbow motion [9–11]. 

Closed reduction and cast immobilisation is generally the treatment of choice for type I Monteggia fracture-dislocations. In one fifth of cases when a closed reduction is performed, reduction may be lost when there is an oblique or comminuted fracture of the ulna [2]. If Monteggia fracture-dislocations are diagnosed and treated acutely, anatomic reduction of the radial head is assured. There are however reports in the literature of soft tissue entrapment impeding reduction. An open approach to reduce the radial head has demonstrated interposition of the radial nerve [12], annular ligament [13], and biceps tendon [14]. Watson and Singer [15] reported entrapment of the median nerve in a greenstick fracture of the ulna in a six-year-old girl, which prevented reduction. 

Nerve palsies at the time of injury are recognised and are uncommon sequelae of type I Monteggia fracture-dislocations [4, 5]. The posterior interosseous nerve is most commonly injured due to its proximity to the radial head and neck as it enters the supinator muscle through the arcade of Frohse [4]. In children the arcade may be thinner and more pliable, and hence lesions resolve more rapidly than in adults [16]. Normally such palsies are treated expectantly with resolution within 9 weeks and electromyographic return of neurological function within 12 weeks [17]. 

Flexible intramedullary nailing has now become the treatment modality of choice for certain long bone fractures in children. It is a safe method, with shortened operating times, minimal soft tissue dissection, easier implant removal, and excellent cosmesis. Indications include unstable and irreducible fractures, when nonoperative treatment fails and in some special cases such as radial neck fractures [18]. Complications of the use of intramedullary nails in the forearm are extremely infrequent with injury to the superficial radial nerve and extensor pollicis longus and brevis rupture due to prominent metalwork reported [19]. 

In our case, an elastic stable intramedullary nail was used to maintain reduction in this unstable type I Monteggia fracture-dislocation. Manipulation of the forearm at the time of nail insertion or when the nail was traversing the fracture site may have displaced the butterfly fragment in such a way as to abut the median nerve and later cause sensory deficit in the thumb, index, and middle fingers. Alternatively the fragment may have further displaced following discharge as the patient was in a lightweight splint, which did not completely immobilise the forearm. As preoperatively there was no neurovascular deficit, we felt that appropriate investigation with electromyography and magnetic resonance imaging was needed to identify the site of the median nerve lesion to allow for planning of early median nerve exploration. There is great potential for bone remodelling in children as seen from our patient's radiographs taken six months after the injury (Figure 4); however this should not be always relied upon as initial good reduction and stabilisation are necessary in some circumstances. Nerve palsies occurring at the time of injury tend to resolve without intervention in the great majority of cases [4, 5, 16]. However in the case presented here symptoms developed following surgical intervention to reduce and fix the fracture. This should alert the surgeon to the possibility of an iatrogenic lesion, the consequences of which can be minimised by expedient recognition, investigation, and treatment [20].

## Figures and Tables

**Table 1:** Bado's classification of true Monteggia fracture-dislocations [8].

Type	Dislocation of radial head	Fracture
I	Anterior	Ulna diaphysis
II	Posterior	Ulna diaphysis
III	Lateral	Ulna metaphysis
IV	Anterior	Proximal ulna and radius diaphysis
